# Supplementary material for: Iterative improvement in the automatic modular design of robot swarms
Source: PeerJ Comput Sci. 2020 Dec 7;6:e322. doi: 10.7717/peerj-cs.322 (PMC7924708; doi:10.7717/peerj-cs.322)
Supplement: Supplemental Information 3 [file peerj-cs-06-322-s003.zip › argos3/doc/api/standalone/a00647_source.html]

ARGoS: plugins/simulator/visualizations/qt-opengl/qtopengl\_lua\_main\_window.h Source File


- Main Page
- Related Pages
- Namespaces
- Classes
- Files

- File List
- File Members

# plugins/simulator/visualizations/qt-opengl/qtopengl\_lua\_main\_window.h

Go to the documentation of this file.

```
00001 
00007 #ifndef QTOPENGL_LUA_MAIN_WINDOW_H
00008 #define QTOPENGL_LUA_MAIN_WINDOW_H
00009 
00010 namespace argos {
00011    class CQTOpenGLLuaMainWindow;
00012    class CQTOpenGLLuaEditor;
00013    class CQTOpenGLLuaFindDialog;
00014    class CQTOpenGLMainWindow;
00015    class CLuaController;
00016    class CComposableEntity;
00017 }
00018 
00019 class QAction;
00020 class QStatusBar;
00021 class QTableWidget;
00022 class QTreeView;
00023 
00024 #include <QMainWindow>
00025 
00026 namespace argos {
00027 
00028    class CQTOpenGLLuaMainWindow : public QMainWindow {
00029 
00030       Q_OBJECT
00031 
00032    public:
00033 
00034       CQTOpenGLLuaMainWindow(CQTOpenGLMainWindow* pc_parent);
00035       virtual ~CQTOpenGLLuaMainWindow();
00036 
00037    public slots:
00038 
00039       void New();
00040       void Open();
00041       void OpenRecentFile();
00042       bool Save();
00043       bool SaveAs();
00044       void Execute();
00045       void Find();
00046       void CodeModified();
00047       void CheckLuaStatus(int n_step);
00048       void HandleMsgTableSelection();
00049       void HandleEntitySelection(size_t un_index);
00050       void HandleEntityDeselection(size_t);
00051       void VariableTreeChanged();
00052       void FunctionTreeChanged();
00053 
00054    private:
00055 
00056       bool MaybeSave();
00057       void PopulateLuaControllers();
00058       void ReadSettings();
00059       void WriteSettings();
00060       void CreateCodeEditor();
00061       void CreateLuaMessageTable();
00062       void CreateLuaStateDocks();
00063       void CreateFileActions();
00064       void CreateEditActions();
00065       void CreateCodeActions();
00066       void OpenFile(const QString& str_path = QString());
00067       bool SaveFile(const QString& str_path = QString());
00068       void SetCurrentFile(const QString& str_path);
00069       void UpdateRecentFiles();
00070       void SetMessage(int n_row,
00071                       const QString& str_robot_id,
00072                       const QString& str_message);
00073 
00074       QString StrippedFileName(const QString& str_path);
00075 
00076       virtual void closeEvent(QCloseEvent* pc_event);
00077 
00078    private:
00079 
00080       enum { MAX_RECENT_FILES = 5 };
00081 
00082       CQTOpenGLMainWindow* m_pcMainWindow;
00083       QStatusBar* m_pcStatusbar;
00084       CQTOpenGLLuaEditor* m_pcCodeEditor;
00085       CQTOpenGLLuaFindDialog* m_pcFindDialog;
00086       QDockWidget* m_pcLuaMsgDock;
00087       QTableWidget* m_pcLuaMessageTable;
00088       QDockWidget* m_pcLuaVariableDock;
00089       QDockWidget* m_pcLuaFunctionDock;
00090       QTreeView* m_pcLuaVariableTree;
00091       QTreeView* m_pcLuaFunctionTree;
00092 
00093       std::vector<CLuaController*> m_vecControllers;
00094       std::vector<CComposableEntity*> m_vecRobots;
00095       size_t m_unSelectedRobot;
00096       QString m_strFileName;
00097 
00098       QAction* m_pcFileNewAction;
00099       QAction* m_pcFileOpenAction;
00100       QAction* m_pcFileOpenRecentAction[MAX_RECENT_FILES];
00101       QAction* m_pcFileSaveAction;
00102       QAction* m_pcFileSaveAsAction;
00103       QAction* m_pcFileSeparateRecentAction;
00104       QAction* m_pcEditUndoAction;
00105       QAction* m_pcEditRedoAction;
00106       QAction* m_pcEditCopyAction;
00107       QAction* m_pcEditCutAction;
00108       QAction* m_pcEditPasteAction;
00109       QAction* m_pcEditFindAction;
00110       QAction* m_pcCodeExecuteAction;
00111 
00112    };
00113 
00114 }
00115 
00116 #endif
```

---

Generated on 10 Jul 2018 for ARGoS by 
 1.6.1
